# Hemodynamic Monitoring in Sepsis—A Conceptual Framework of Macro- and Microcirculatory Alterations

**DOI:** 10.3390/diagnostics11091559

**Published:** 2021-08-28

**Authors:** Liana Valeanu, Serban-Ion Bubenek-Turconi, Carmen Ginghina, Cosmin Balan

**Affiliations:** 1 1st Department of Cardiovascular Anesthesiology and Intensive Care, “Prof. C. C. Iliescu” Emergency Institute for Cardiovascular Diseases, 258 Fundeni Road, 022328 Bucharest, Romania; liana.valeanu@yahoo.com (L.V.); bubenek@alsys.ro (S.-I.B.-T.); 2 Department of Anesthesiology and Intensive Care, University of Medicine and Pharmacy “Carol Davila”, 8 Eroii Sanitari Blvd, 050474 Bucharest, Romania; 3 3rd Department of Cardiology, “Prof. C. C. Iliescu” Emergency Institute for Cardiovascular Diseases, 258 Fundeni Road, 022328 Bucharest, Romania; carmenginghina2010@gmail.com; 4 Department of Cardiology, University of Medicine and Pharmacy “Carol Davila”, 8 Eroii Sanitari Blvd, 050474 Bucharest, Romania

**Keywords:** sepsis, shock, hemodynamic, monitoring, macrocirculation, microcirculation

## Abstract

Circulatory failure in sepsis is common and places a considerable burden on healthcare systems. It is associated with an increased likelihood of mortality, and timely recognition is a prerequisite to ensure optimum results. While there is consensus that aggressive source control, adequate antimicrobial therapy and hemodynamic management constitute crucial determinants of outcome, discussion remains about the best way to achieve each of these core principles. Sound cardiovascular support rests on tailored fluid resuscitation and vasopressor therapy. To this end, an overarching framework to improve cardiovascular dynamics has been a recurring theme in modern critical care. The object of this review is to examine the nature of one such framework that acknowledges the growing importance of adaptive hemodynamic support combining macro- and microhemodynamic variables to produce adequate tissue perfusion.

## 1. Introduction

The key pillars of sepsis management are source control, antimicrobial therapy, and circulatory resuscitation [1]. The deliberate and early recognition of sepsis, especially when hemodynamic instability occurs, constitutes the element that confers the weightiest survival benefit [2]. Optimal hemodynamic resuscitation represents a subject of intense debate believed to improve outcomes further [3].

A myriad of mechanisms combines to produce hypotension and impair end-organ perfusion in sepsis. Their relative contribution is time-dependent and exhibits intra- and inter-individual variance based on a unique set of host risk factors and immune response. Macro- and microcirculatory disturbances are equally meaningful and can be lumped pragmatically into two categories: peripheral vascular dysfunction and myocardial dysfunction. The former includes venous and arterial vasodilation, compromised microcirculatory flow distribution (i.e., the coexistence of stopped-, normal-, intermittent-, and high-flow capillary units) [4], and ubiquitous shock-induced endotheliopathy (SHINE) with damaged glycocalyx and increased capillary permeability [5]. The latter has been traditionally attributed to left ventricular (LV) systolic dysfunction. However, increasing evidence supports the right ventricle (RV) role that, far from being a mere passive conduit of the heart, may become impaired and limit LV performance [6]. Furthermore, diastolic dysfunction assumes more credit as it is increasingly evident that it can deleteriously affect outcomes by itself [7]. Altogether, these circulatory derangements constitute the basis for an altered hemodynamic state mechanistically characterized by potentially disrupted ventricular interdependence, right and left ventricular–arterial (VA) uncoupling [8], inactivation of vascular waterfalls (i.e., precapillary Starling resistor that generates a pressure gradient between Permutt and Riley’s arterial critical closing pressure (CCP) and Guyton’s mean systemic filling pressure (MSFP) meant to stabilize tissue perfusion should low blood flow ensue) [9,10,11], and loss of hemodynamic coherence [12]. In accord with this precept, protocolized care has shifted from a “one size fits all” paradigm to an individualized framework that endorses specific hemodynamic targets, adaptive multi-parametric monitoring, and functional assessment of the cardiovascular reserve to ensure the adequacy of end-organ blood flow [13].

The scope of this review is to delineate and discuss proposed components of this contemporary physiology-based “goal-directed” management of circulatory disturbances. Emphasis is placed on discriminating macro- and microcirculatory endpoints and how both components could be reconciled and combined to optimize tissue perfusion.

## 2. Macrocirculation

Traditionally, hemodynamic resuscitation has aimed to prevent or revert tissue hypoxia by improving a range of macrocirculatory endpoints. The inherent assumption of this approach is a linear relationship between macrohemodynamics and end-organ hypoxia, a cellular phenomenon. To some extent, this may be true, especially in the initial stages of sepsis-related acute circulatory dysfunction. Nonetheless, progressive stages exhibit both endothelial dysfunction and cytopathic hypoxia [14]. The former marks a new circulatory status where macrocirculation and microcirculation become uncoupled (i.e., hemodynamic incoherence), meaning that tissue perfusion may not improve or may even worsen after a systemic flow increase [15]. The latter potentially signals a second level of uncoupling between the microcirculation and the mitochondrial respiratory chain complexes. Thus, by and large, securing macrocirculation integrity seems imperative but not always sufficient to guarantee adequate tissue oxygen tension.

### 2.1. Resuscitation Endpoints

#### 2.1.1. Blood Pressure

Blood pressure-driven resuscitations constitute the norm. Nonetheless, multiple pathophysiological disturbances contribute to generating the blood pressure signal. Therefore, root cause analysis of hypotension is a prerequisite to defining treatment rationally (see Section 2.2).

Defining hypotension at the bedside may be an elusive task. Provided that the downstream pressure is negligible, the mean arterial pressure (MAP) equates to organ perfusion pressure, and the recommended initial target is a MAP of 65 mmHg [1]. In actual practice, healthcare professionals often push MAP well above 65 mmHg [16,17]. Whether this approach benefits or harms patients remains debatable. A recent retrospective analysis of 8782 patients found heightened risks for mortality, acute kidney injury (AKI), and myocardial injury at MAP thresholds lower than 85 mmHg [18]. Contrastingly, a pooled analysis of two major trials, SEPSISPAM and OVATION, comparing higher versus lower MAP targets, found that higher thresholds associated with the use of vasopressors for more than 6 h before randomization may increase mortality (odds ratio (OR), 3.00; 95% CI, 1.33–6.74; *p* = 0.017) [19]. Notably, SEPSISPAM showed that higher MAP among patients with chronic hypertension reduced the need for renal replacement therapy, albeit a difference in mortality was absent; whereas, among all patients, it led to an increased rate of atrial fibrillation [16]. Lastly, Lamontagne et al. randomized 2600 patients with vasodilatory hypotension aged 65 years or older to permissive hypotension (MAP 60–65 mmHg) or usual care and reported no difference in 90-day all-cause mortality between the study groups (41% permissive hypotension versus 43.8% usual care, absolute risk reduction (ARR), −2.85%; 95% CI, −6.75 to 1.05; *p* = 0.15) [20].

Abnormal downstream pressures are common in the critically ill population. In such cases, the pressure difference between upstream (i.e., MAP) and downstream pressures (e.g., IAP—intraabdominal pressure, ICP—intracranial pressure or CVP—central venous pressure, whichever is higher) must be considered to preserve organ perfusion pressure. Mean perfusion pressure (MPP), defined as the pressure difference between MAP and CVP, is a physiological construct that attempts to approach intrarenal hemodynamics. Systemic venous congestion (i.e., high CVP) emerged as a significant contributor to acute kidney injury (AKI) pathophysiology, first in patients with acute and chronic cardiac disease [21], and then in critically ill patients with sepsis [22]. One study looking at patients with septic AKI found that the MPP deficit (i.e., the difference between pre-morbid MPP values and those achieved during resuscitation) was common despite NE administration, higher among those with septic shock and severe AKI and mainly driven by CVP excess. Interestingly, CVP was independently associated with worsening AKI (adjusted OR, 1.26; 95% CI, 1.03–1.51), whereas MPP deficit was not [23]. Another study reported MPP lower than 60 mmHg to be independently associated with AKI progression, and authors emphasized that elevated CVP impacted kidney outcome more than MAP [24].

Some relevant information can be gleaned from these studies. Firstly, MAP goals are not to be assumed a priori but should be sought for each patient. To this end, a vasopressor challenge may be transiently applied to meet resuscitation goals, with a return to baseline dosing in case targets remain abnormal at higher MAP [25]. Secondly, because response assessment after any therapeutic attempt is crucial, several physiological read-outs should be subsumed to provide reliable feedback (e.g., lactate, capillary refill time (CRT), urine output, and level of consciousness). Thirdly, for the same MAP, systemic (i.e., high CVP) or regional (i.e., high IAP or ICP) congestion, if overlooked, is likely to impair organ-specific perfusion.

Metrics other than MAP could provide helpful guidance as well. Loss of vascular tone, both arterial and venous, is a quintessential component of sepsis-related circulatory dysfunction. Therefore, early initiation of vasopressors to set the venous tone and reinstate adequate venous return was deemed teleologically reasonable [26]. Indeed, experimental sepsis and clinical data have indicated that, compared to fluid resuscitation alone or late start of vasopressors, an early start of norepinephrine (NE) combined with fluid resuscitation could help limit volume requirements and time until shock control, and be associated with improved tissue oxygenation, superior splanchnic blood flow redistribution, and better clinical outcomes [27,28,29]. Conclusively, hypotension with a low diastolic arterial pressure (DAP) (i.e., <40 mmHg), particularly in the case of tachycardia, strongly indicates reduced vascular tone and urges immediate administration of NE even before fluid repletion is complete [30]. In this respect, the diastolic shock index (DSI), calculated as heart rate (HR) divided by DAP, may be a promising tool still awaiting confirmation that could help signal severe vasoplegia [31]. Conceivably, DSI could then be used to initiate fluid sparing strategies in patients most likely to benefit, i.e., the predominantly vasoplegic phenotype.

Lastly, pulse pressure (PP) performs as a qualitative proxy of stroke volume (SV) [32]. More than an isolated value, its trend over time offers an immediate window to heart function yet does not absolve from more extensive monitoring of cardiac pathology and cardiovascular functional reserve.

#### 2.1.2. Flow

Optimization rather than maximization of oxygen delivery to tissues, and implicitly its primary determinant—cardiac output (CO), is the mainstay to treating circulatory failure. Simultaneously evaluating more than one variable is essential to understand the hemodynamic state, but redundancy must be avoided at all costs to facilitate timely and meaningful decision-making at the bedside. Is CO monitoring relevant for daily clinical practice?

To answer, it is worth considering matters from a thermodynamic perspective. Perfusion pressure (i.e., MAP) is an intensive property of the circulatory system, meaning that it is independent of the size of the system, whereas CO is an extensive (or capacitive) property, dependent on the size of the system. This aspect, coupled with markedly different metabolic needs and vasomotor tone among patients, explains why a narrow range of effective perfusion pressures (e.g., 60–85 mmHg) results in a highly variable CO both inter- and intra-individually. Consequently, CO falls short of qualifying as a chief resuscitation endpoint [33]. Nevertheless, CO monitoring remains an excellent indicator of responses to treatment (e.g., inodilators) and is required to understand the hemodynamic phenotype (e.g., a distributive shock) or any of its fluctuations [34]. A failure to improve clinical outcomes [35] is not a conclusive argument to discard CO monitoring as this instead reflects the need to revise treatment choices [36]. Also, the same instruments used to compute and measure CO can serve to assess essential hemodynamic principles (see Section 2.3).

**Figure 1:**
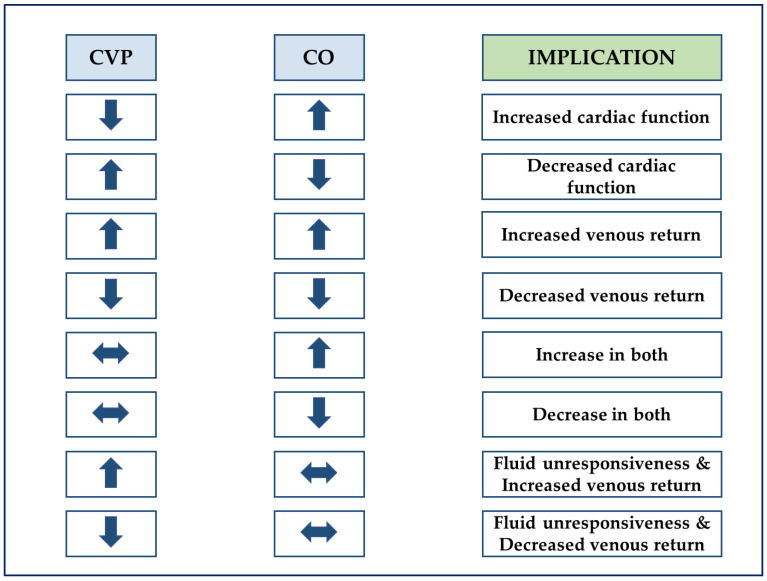
Interpretation of coupled changes in cardiac output and central venous pressure. CO, cardiac output; CVP, central venous pressure. Adapted with permission [37].

Changes in CO yield valuable insights, especially when coupled with changes in CVP. Joint consideration of both variables informs on the adequacy of cardiac function and venous return (see Figure 1) [37] and was recently proposed to set the rate of fluid removal during volume depletion phases (i.e., using either ultrafiltration or diuretics). In this last respect, close monitoring of tolerance (i.e., CO is maintained) and efficacy (i.e., CVP is decreased) could enable the fine-tuning of fluid removal to closely match the interstitial–vascular refill rate [38].

#### 2.1.3. Tissue Perfusion

Macrocirculatory or global markers of tissue perfusion are predefined circulatory endpoints, constituting the basis of quantitative resuscitation strategies (i.e., goal-directed therapies (GDT)), and their early application reduce mortality compared to qualitative strategies [39]. Three main tools are currently in use: venous oxygen saturations, either central (i.e., ScvO_2_) or mixed (i.e., SmvO_2_), lactate, and carbon dioxide (CO_2_) gaps.

Following the successful Rivers’ Early Goal-Directed Therapy (EGDT) trial [40], three independent randomized controlled trials (RCT) comparing ScvO_2_-driven protocols to usual care failed to replicate the original findings and questioned the validity of ScvO_2_ as a resuscitation endpoint [41,42,43]. However, the interventional strategies caused no harm. Compared to the original EGDT, all three trials appear to have included less severe patients given lower baseline lactate and ScvO_2_ greater or equal to 70% [44]. Of note, cytopathic hypoxia, characteristically associated with sepsis, precludes the indiscriminate use of ScvO_2_ to monitor the O_2_ supply/demand balance (DO_2_/VO_2_) [45]. To affect outcomes, choosing the right tool for the right patient seems decisive.

Measurement of lactate is pivotal to monitor and guide therapy in all forms of shock. There are several causes of hyperlactatemia in sepsis, including tissue hypoxia, increased glycolysis, adrenergic stimulation, pyruvate dehydrogenase inhibition, and altered clearance [46]. Regardless of the cause, hyperlactatemia is consistently associated with severity of illness and prognosis [47]. During acute changes with therapy or disease progression, lactate kinetics typically lag behind other metrics such as ScvO_2_ and CO_2_ gaps. Thus, the need to combine several endpoints is fundamentally linked to this aspect.

CO_2_ gaps include the measurement of venous-to-arterial CO_2_ partial pressure difference (PvaCO_2_) or the calculation of the venous-to-arterial CO_2_ content difference (CavCO_2_), but this last variable is cumbersome to apply in daily routine. In addition, mixed and central venous blood are interchangeable, so the minimum set up to perform this type of monitoring is a central venous catheter and an arterial line [48].

Detailed analysis of CO2-derived variables can be found elsewhere [49]. In brief, PvaCO2 performs well as a marker of the adequacy of CO for a given metabolic condition (i.e., PvaCO2 × CO ≈ CO2 production (VCO2)) [50]. Furthermore, reflection of tissue hypoxia is possible when PvaCO2 is indexed to the arterio–venous oxygen content difference (CavO2) [51]. Interestingly, Ospina-Tascón et al. found PvaCO2 closely correlated with microvascular blood flow and unrelated to any global hemodynamic variables [52]. If that were confirmed, an increase in CO that elicits no change in PvaCO2 could represent a valuable signal of hemodynamic incoherence.

**Figure 2:**
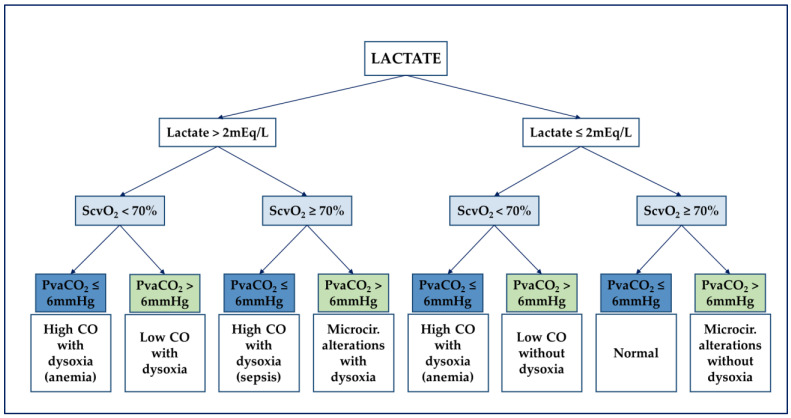
Flow chart for analyzing the hemodynamic profile according to De Backer. ScvO_2_, central venous oxygen saturation; CO, cardiac output; Microcir, microcirculatory; PvaCO_2_, venous-to-arterial carbon dioxide partial pressure difference. Reproduced with permission [54].

CO2 gaps address many of the barriers associated with previously described markers. Unlike lactate, PvaCO2 provides real-time bedside feedback, and, unlike ScvO2, it remains informative in the case of sepsis-driven cytopathic hypoxia [53]. In combination, these variables assemble into a three-tier structured approach to identify and discriminate macro- and microcirculatory disturbances (see Figure 2) [54].

### 2.2. Macrocirculation—Monitoring Toolkit

Ultrasound (US) has developed into a holistic tool that evaluates nearly all organ systems (i.e., heart, lungs, vessels, abdomen, and brain) [55,56]. In addition, there is an increasing amount of evidence to suggest that US is particularly well suited to run personalized management of critically unwell patients. For example, a combination of echocardiographic and clinical data was recently shown to identify five distinct macrocirculatory phenotypes in septic shock, including “well-resuscitated”, “still-hypovolemic”, hyperkinetic, LV systolic dysfunction, and RV failure, with LV diastolic dysfunction contributing equally across the last four phenotypes [57]. Each hemodynamic profile carries a specific risk of morbidity and mortality, which undifferentiated management would most likely aggravate. US may even extend over macrocirculation to further characterize regional blood flow in the kidney, liver, and spleen (see Section 2.3.2). [58].

Accordingly, echocardiography (i.e., either transthoracic (TTE) or transesophageal (TEE)) is accepted today as the first-line modality to assess patients with circulatory failure [59,60]. The minimal hemodynamic toolkit consists of an arterial line, a central venous line, and a US machine. Although this minimal toolkit provides answers to many clinically consequential questions regarding cardiac pathology and cardiovascular performance (see Appendix A, Table A1 and Section 2.3), some gaps are still left to be filled. In those cases, the pulmonary artery catheter (PAC) and transpulmonary thermodilution (TPT) would provide additional data on circulatory pressures and volumes, enhancing the overall diagnostic yield.

Both PAC (i.e., fast-response volumetric PAC) and TPT allow for continuous tracking of CO variations, proving particularly useful to test fluid responsiveness, spot circulatory events early even before hypotension ensues and closely monitor changes in therapy (e.g., inotrope or vasopressor administration, fluid challenge). Furthermore, PAC also provides precise monitoring of pulmonary arterial pressure (PAP) and left atrial pressure (LAP) (as pulmonary artery wedge pressure, PAWP) and even a model-driven estimation of pulmonary capillary pressure (PCP) based on mono- or biexponential fitting of the PAP transient after balloon inflation [61]. On the other hand, TPT provides insight on extravascular lung water (EVLW) and pulmonary vascular permeability (PVPI), an index of pulmonary capillary leak. These features specifically advocate PAC in difficult to treat cases of RV failure or acute cor pulmonale (ACP) and TPT in moderate-to-severe acute respiratory distress syndrome (ARDS), the choice of one over the other mainly depending on clinical priorities. Nevertheless, compared to echocardiography, neither PAC nor TPT can identify the distinct cardiovascular components, functional and structural, generating CO, some of which constitute potential sources of error for thermodilution-based techniques (e.g., intracardiac shunts, severe tricuspid or pulmonic valve regurgitation, or severe left-sided regurgitations for TPT only). Spectral doppler interrogation of blood flow and myocardial velocities allows a punctual but comprehensive and non-invasive functional assessment of pressures (e.g., LAP, PAP), CO, systolic and diastolic function, and coupling of the heart with circulation. Overall, it appears that echocardiography in shock, although self-sufficient on many occasions and indispensable on most, is empowered by invasive methods in complex scenarios [62].

### 2.3. Hemodynamic Principles

#### 2.3.1. Fluid Responsiveness

Fluid responsiveness (or preload dependence) is a predefined increase in SV after a predetermined increase in preload. Prediction of fluid responsiveness is mandatory for several reasons. Firstly, volume and sodium overload have been consistently associated with worsened outcomes and less than 40% of hypotensive patients with sepsis are fluid responders [63]. Secondly, infringement of this first principle may precipitate a paradoxical decrease in oxygen delivery (DO_2_) and negate the very essence of volume expansion [64]. Equally important, confirmation of preload independence may help tailor ultrafiltration rates and prevent cardiovascular instability in patients under continuous renal replacement therapy (CRRT) [65,66].

Currently, dynamic parameters to detect preload dependence are unanimously recommended over static markers of cardiac preload. Using US, one such parameter is the variation in left-ventricular outflow tract (LVOT) volume-time integral (VTI) either with respiration or after a passive-leg raising (PLR) test. Considering a series of strict preconditions to be met with the former (e.g., regular heart rhythm, mechanical ventilation with at least 8 mL/kg tidal volume and no spontaneous breaths, closed-chest, normal chest wall/lung elastance ratio, and heart rate/respiratory rate ratio >3.6) [67], testing with PLR proves more feasible, especially within the intensive care unit (ICU) [68]. Other preload changers, such as the end-expiratory occlusion test, alone or in combination with end-inspiratory holds, or the “mini-fluid” challenge, constitute practical alternatives that efficiently circumvent PLR limitations (e.g., elevated IAP or ICP, lower limb trauma) [69].

To ensure the proper application of the first principle, several aspects merit consideration. Firstly, fluid responsiveness is intrinsically linked to normal physiological conditions and, as a corollary, fluid unresponsiveness is always pathological, regardless of whether it is spontaneous or iatrogenic. The latter instance typically stems from the complete utilization of preload reserve to maximize end-organ blood flow. As recently suggested by experimental data, this traditional practice forces an unphysiological state, may cause harm and should be discarded in favor of an individualized fluid therapy running on the steep part of the Frank–Starling curve [70,71]. Secondly, dynamic indices to assess preload status provide a dichotomous outcome of responsive versus non-responsive, but this hardly mirrors the bedside reality. Indeed, almost a quarter of patients may lie in a “grey zone” where preload dependence cannot be predicted reliably [72]. Such cases demand that further corroborating evidence be obtained before fluid loading, preferably from tests with high specificity [73,74]. Thirdly, prediction of fluid responsiveness allows no assumptions regarding the safety, longevity of intravascular response, and optimum rate (e.g., bolus versus continuous) of fluid administration. Lastly, macrocirculatory responsiveness does not guarantee microcirculatory responsiveness, hence the importance of incorporating microcirculatory variables into clinical practice [15].

#### 2.3.2. Fluid Tolerance

**Figure 3:**
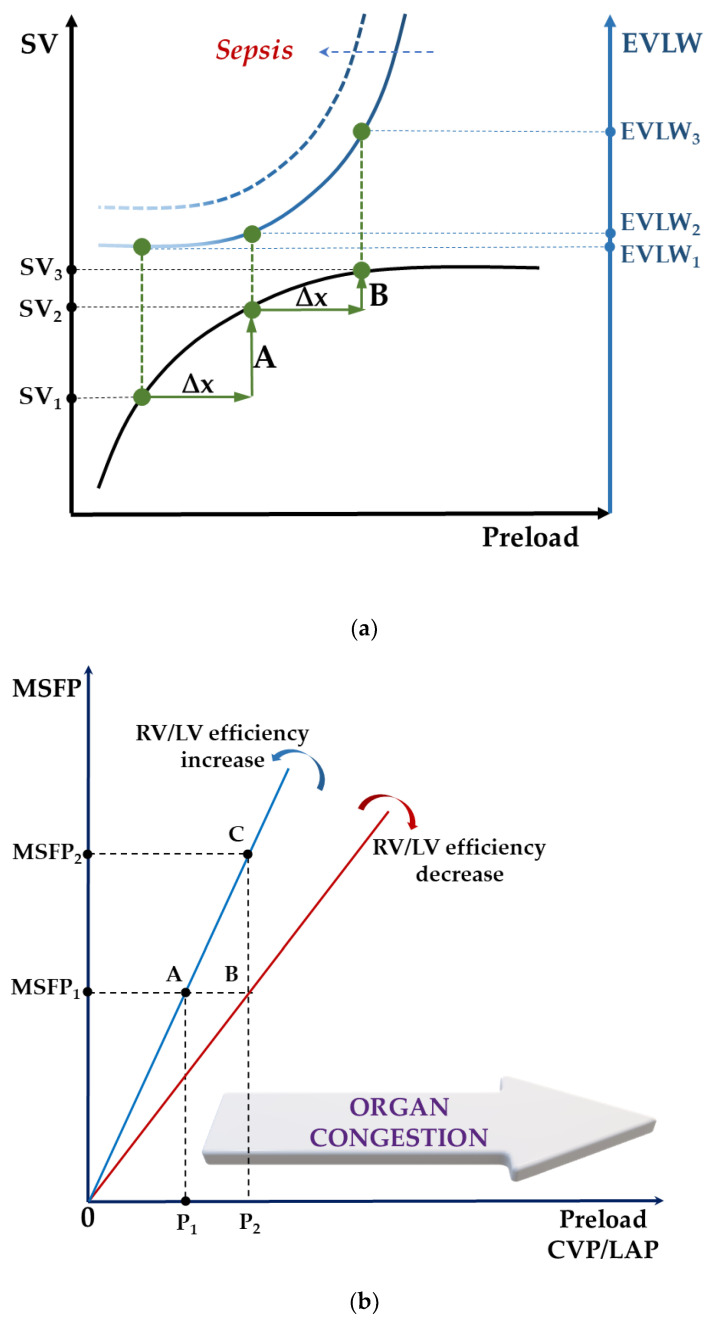
(**a**) A schematic illustration of superimposed Frank–Starling (black) and Marik–Phillips (solid blue) curves demonstrating the effects of an identical preload challenge (∆x) on SV and EVLW in a preload-dependent (A) and preload-independent state (B). A: a steep increase in SV (SV_1_ → SV_2_) with minimal increase in EVLW (EVLW_1_ → EVLW_2_); B: a minimal increase in SV (SV_2_ → SV_3_) with a steep increase in EVLW (EVLW_2_ → EVLW_3_). Sepsis alters the capillary permeability resulting in a leftward shift of the EVLW curve (dotted blue). EVLW, extravascular lung water; SV, stroke volume; ∆x, a specific preload challenge. Adapted with permission [75]. (**b**) A schematic illustration of how ventricular performance (i.e., global RV/LV efficiency) and volume state (i.e., MSFP) interact to produce either increased CVP resulting in extrathoracic congestion (e.g., liver, kidney, mesenteric), increased LAP resulting in pulmonary edema, or a mixture of both. Increased permeability independently aggravates tissue congestion. Decompartmentalization occurs in severe conditions, resulting in generalized edema. A to B: for the same volume state, decreased RV/LV efficiency risks fluid intolerance. A to C: preserved RV/LV efficiency does not guarantee fluid tolerance with fluid loading. As a corollary, a normal volume state (i.e., MSFP) does not ensure fluid tolerance in case of severely impaired RV/LV efficiency. Increased CVP could also result from an altered Ecw/El ratio (e.g., intra-abdominal hypertension). CVP, central venous pressure; Ecw, chest wall elastance; El, lung elastance; LAP, left atrial pressure; LV, left ventricle; MSFP, mean systemic filling pressure; RV, right ventricle.

Tissue edema and fluid loading are invariably interlinked (see Figure 3a) [75], which means that, despite fluid responsiveness, tolerance to further volume may be jeopardized because of elevated capillary filtration pressures, increased endothelial permeability, or both. Conversely, in true fluid responders, fluid boosts DO2 without causing lung edema, significant hemodilution or RV dysfunction with increased CVP and subsequent end-organ congestion (see Figure 3b). Accordingly, assessment of fluid tolerance is also essential to remove fluids.

US is an excellent tool to test fluid tolerance non-invasively at the bedside. For example, lung sonographic B-lines and Doppler-estimated LAP can be combined to detect and discriminate cardiac from non-cardiac causes of excess EVLW [76]. In addition, the venous excess US (VExUS) score is a recently proposed four-tier protocol that grades venous congestion in the inferior vena cava (IVC) and three target organs, including the liver (i.e., hepatic veins), gut (i.e., portal veins), and kidneys (i.e., intrarenal veins) [77].

The clinical impact of venous congestion is much more substantial than previously considered. This is best appreciated by realizing that end-organ flow, simplistically assigned to MAP minus CVP (i.e., MPP), actually runs in a “vascular bottleneck” between the precapillary arterioles and postcapillary venules [78]. Consequently, within this tight microvascular pressure gradient, venous pressure rises are bound to alter tissue perfusion more than MPP predicted and eventually risk “microcirculatory tamponades” [79]. Optimizing venous pressures thus becomes both a macro- and microcirculatory priority.

#### 2.3.3. Ventricular–Arterial Coupling

**Figure 4:**
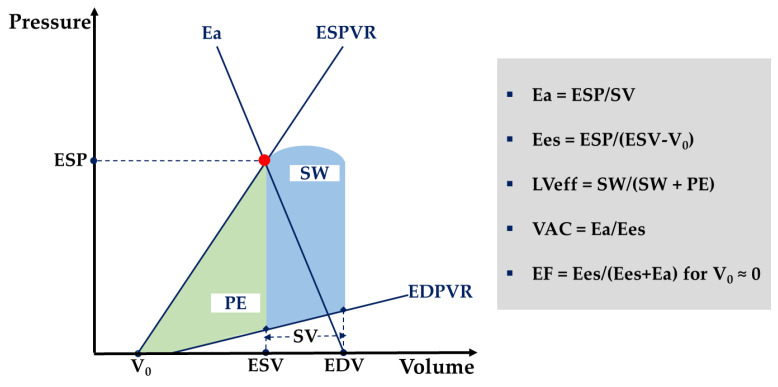
A schematic illustration of ventricular–arterial coupling (VAC). The left ventricle (LV) is characterized by the end-systolic and end-diastolic pressure–volume relationship (ESPVR and EDPVR). The end-systolic LV elastance (Ees) is the slope of the ESPVR line. V_0_, the LV end-systolic unstressed volume, is the intercept of ESPVR with the volume axis. The arterial system is characterized by the arterial elastance (Ea), i.e., the slope of the Ea line that connects the end-diastolic volume (EDV) with the end-systolic point (red dot). End-systolic coordinates are the end-systolic pressure (ESP) and the end-systolic volume (EDV). Stroke work (SW) (blue area) is maximum for Ea/Ees of 1 and a corresponding ejection fraction (EF) of 50%. LV metabolic efficiency (LVeff) is maximum for Ea/Ees close to 0.5 and a corresponding EF of 66%. PE, end-systolic potential energy (green area). Adapted after Hayashida et al. [80].

The same cardiac output may virtually result from infinite combinations of LV contractility and loading conditions. However, from a myocardial energetics perspective, only a particular combination will provide the optimum ventricular energy conversion and transmission towards the arterial system [80]. This ventricular–arterial interaction, commonly referred to as VA coupling, is mathematically represented by the ratio of the systemic arterial elastance (i.e., Ea—a combined measure of arterial load exerted on the LV, dependent on arterial resistance and compliance, heart rate, and aortic impedance) to the LV end-systolic elastance (i.e., Ees—a load-independent measure of LV contractility) (see Figure 4). With the advent of Chen’s single beat echocardiography-based method to estimate Ees, monitoring VA coupling (i.e., Ea, Ees, and Ea/Ees) has entered the clinical arena and has since fueled intense research in the critically ill population [81]. VA decoupling, defined as Ea/Ees ratio >1.36, was reportedly common in a series of septic shock patients and resulted from changes in Ees, Ea, or both [82]. Another study found bedside determination of VA coupling to identify, explain, and predict circulatory responses to therapy [83]. These data suggest that sustained VA decoupling is disadvantageous and may result in heart failure, loss of preload recruitability, and eventually poor clinical outcomes. As a direct corollary, therapies that revert VA decoupling are expected to improve metabolic and mechanical cardiovascular efficiency and reduce morbidity and mortality. For instance, Guarracino et al. endorse VA coupling monitoring to fine-tune the administration of inotropes and inodilators, vasopressors and vasodilators, and fluids (see Appendix A, Table A1 and Table A2) [84]. To encourage bedside calculation of VA coupling, the same group of authors have recently released a mobile application (iElastance©) based on Chen’s method [85].

Finally, combined echocardiography and invasive arterial monitoring may provide an additional VA coupling measure (i.e., the dynamic arterial elastance, Eadyn). In contrast to a steady-state index such as Ea, Eadyn is a unitless measure representing the dynamic relationship between the respiratory changes in the arterial PP (PPV) and SV (SVV) (i.e., PPV/SVV). Preliminary reports promoted Eadyn as an index directly proportional to arterial tone [86,87]. Contrarily, the latest research found Eadyn inversely related to vasomotor tone, representing an index of the coupling between the circulation and the heart that moves in the opposite direction to the Ea/Ees ratio [88,89]. Hence, higher Eadyn seemingly signals improved VA coupling (i.e., lower Ea/Ees ratio) instead of an increased vasomotor tone. Eadyn emerged in clinical settings even before the resolution of its true nature. Initially, Eadyn was shown to predict the arterial pressure response after volume in fluid responsive patients [90,91] and later after weaning [92,93] or initiation [83] of norepinephrine irrespective of preload reserve. Altogether, these clinical studies indicate that higher Eadyn puts the cardiovascular system at an advantage, resulting in improved responses to changes in loading conditions. In addition, it is of clinical significance that Eadyn remains valid regardless of breathing pattern, i.e., spontaneous versus controlled (on its application, see Appendix A, Table A1) [94].

Thought-provokingly, Bar and Guinot reported the decrease of Eadyn during norepinephrine infusion to be inversely correlated with the height of the vascular waterfall (i.e., CCP–MSFP), a microvascular phenomenon [95]. This sets the stage for compound indexes reflecting both macro- and microcirculatory alterations.

#### 2.3.4. Volume State Assessment

Recommendations for fluid resuscitation have been ubiquitous across the critical care literature in the latest decade. Still, there has been a surprising lack of consensus on measuring or estimating the intravascular filling status. MSFP, Guyton’s pivotal piece to CO regulation, is one potential method to assess this elusive cardiovascular variable, i.e., the volume state. MSFP equals the stressed blood volume over systemic vascular compliance and changes with absolute volume or capacitance shifts [96].

**Figure 5:**
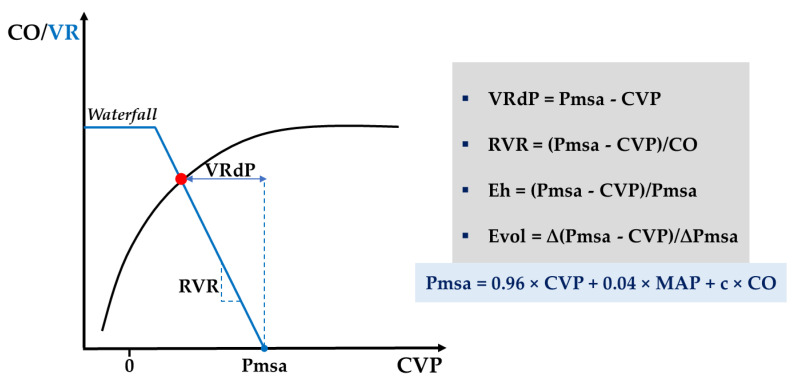
A schematic illustration of the steady-state interaction (red dot) between venous return (blue curve) and cardiac function (black curve) introducing Parkin’s Guytonian perspective on global heart efficiency. c, anthropometric constant; CO, cardiac output; CVP, central venous pressure; Eh, global heart efficiency; Evol, volume efficiency; MAP, mean arterial pressure; Pmsa, mean systemic filling pressure analogue; RVR, resistance to venous return; VR, venous return; VRdP, pressure gradient for venous return; ∆, change after a preload challenge. See Appendix A, Table A1 for further discussion.

Three different bedside estimates of MSFP can similarly track the effective circulatory blood volume [97]. However, the mathematical model developed by Parkin et al. provides an analogue signal of MSFP (Pmsa) that shows the least bias against zero-flow measurements from right atrial balloon occlusions [98]. Additionally, this same mathematical construct provides dimensionless, scalar, and continuous measures to assess the global heart efficiency (Eh) and volume responsiveness (Evol) (see Figure 5 and Appendix A, Table A1). Notably, compared to conventional markers of fluid responsiveness (see Section 2.3.1), Evol assesses the magnitude of response rather than just the presence of response (i.e., responder versus non-responder) after a preload challenge (e.g., PLR test) [99].

There is an ongoing debate about the best way to integrate Pmsa into clinical practice [100]. Conveniently, closed-loop studies suggest that Pmsa provides clinically consequential vector guidance during volume gains or losses [101,102]. Indeed, Pmsa is simply the lumped mathematical equivalent to the split monitoring of CO and CVP proposed by Legrand et al. to guide fluid depletion [38]. Consequently, a stable Pmsa signal during active de-resuscitation (i.e., using diuretics or CRRT) is poised to ensure an adequate balance between fluid removal and vascular refill rates, hence optimum interstitial decongestion with maintained cardiovascular stability.

## 3. Hemodynamic Monitoring of the Microcirculation

### 3.1. The Case for Microcirculatory Assessment

Circulatory homeostasis results from three separate compartments (i.e., macro, micro, and cellular), each under specific laws and regulations that invariably couple and overlap to guard organ function. Preserved in the early phases of shock, this orderly coupling (i.e., hemodynamic coherence) is lost once more advanced tissue or organ damage ensue [12]. Therefore, compartment analysis is conceptually not interchangeable but complementary, hence the imperative to monitor the microcirculatory “black box” itself to resolve coherence. With the advent of handheld vital microscopes (HVM), mounting clinical and experimental evidence has come to support this thesis.

Several mechanisms may render hemodynamic coherence ineffective, preventing the macrocirculation from distributing oxygenated blood to various tissues despite its correction with fluids and vasoactive drugs. In health, microvessels exhibit dense and homogeneous networks, run in the proximity of vascular waterfalls, and obey balanced intrinsic regulation (i.e., myogenic, metabolic, and humoral), ultimately dependent on complex crosstalk between a quiescent endothelium, a “thick” glycocalyx and intact blood rheology [103]. Shock states, including sepsis, were shown to disrupt each of these characteristics. Moreover, therapies aiming to improve macrocirculatory variables may equally benefit and harm microcirculation, an outcome that evades predictability.

The effects of fluid therapy depend on several factors, including timing, type, rate, duration, and amount of fluid. Early but not late sepsis showed marked microcirculatory improvement following fluid administration, a result that remained independent of the type of fluid (i.e., crystalloid versus 4% albumin) and global circulatory effects [104]. Two other studies looked at the macro-microcirculatory coherence following a volume challenge in the early sepsis and showed conflicting results, probably reflecting patients with different severity of illness [15,105].

Fluid overload promotes tissue edema and hampers normal oxygen diffusion. Additionally, secondary hemodilution reduces capillary hematocrit and alters the rheological blood profile resulting in decreased viscosity and abated shear stress-mediated vasoregulation [106]. Red blood cell (RBC) transfusion may thus appear as the ideal candidate in states with low oxygen-carrying capacity, but the available evidence supports a more nuanced view. Sakr et al. found a dichotomous response after RBC transfusion in sepsis, with microcirculatory improvement in patients with altered capillary perfusion at baseline and deterioration in patients with normal baseline [107]. Most likely, rheology also plays a critical role in these observations. Blood viscosity was shown experimentally to override blood oxygen-carrying capacity in maintaining microcirculatory perfusion during normovolemic anemia [106]. Elsewhere, high viscosity plasma was associated with increased perivascular nitric oxide (NO) concentration and vasodilation during hemodilution [108] and elevated functional capillary density (FCD) during hemorrhagic hypovolemia [109]. However, translating these findings into practice would be challenging because of a highly variable basal level in blood viscosity amongst patients due to chronic preexisting conditions.

Compared to crystalloids, albumin and other highly viscous compounds provide a more lasting microcirculatory recruitment with lesser capillary leakage but only within microcirculatory-targeted resuscitative strategies [110].

Conflicting responses to inodilators and vasopressors are commonly reported and may again reflect different dosing or microvascular conditions at baseline [111]. Conversely, selective β1-blockade restored renal vascular waterfalls in an experimental sepsis model, reinforcing the concept that therapies should aim to mitigate SHINE for improved outcomes [10].

Largely independent of the macrocirculatory profile, misaligned treatment choices and shock combined can produce four types of microcirculatory alterations. These may often concur in states of intricate pathogenesis such as sepsis, but one often prevails over the other. They are defined according to Ince et al. as: type 1, complete stagnated capillaries (circulatory arrest, excessive dosing of vasopressors); type 2, reduction in number of capillaries with continuous flow (hemodilution); type 3, plugged capillaries in the vicinity of flowing units (sepsis, hemorrhage); type 4, hyperdynamic capillary flow (sepsis and hemodilution) [112]. This classification constitutes a normative foundation for further research and is also clinically informative, upholding the tenet that optimal therapy must include a microcirculatory feedback loop.

### 3.2. Microcirculation—Monitoring Toolkit

The gold standard (i.e., HVM) and several other methods emerged in the latest decades to expose the microcirculatory compartment for experimental analysis and understanding, raising awareness amongst clinicians.

Originating in intravital microscopy, direct exploration of capillaries evolved from orthogonal polarization spectral (OPS) imaging to sidestream dark field (SDF) imaging and, lately, third generation HVM incorporating incident dark field imaging (IDF). However, most research has focused on the sublingual microcirculatory bed as it is readily accessible and representative of microcirculatory disturbances in other organs [103]. Using hemoglobin-specific wavelength light, capillaries unfold as they border black/gray RBCs on a white background. The presence or lack of RBCs flow differentiates between functional and nonfunctional units, providing a split view on convective and diffusive components based on several datasets of microvascular flow (e.g., microcirculatory flow index, MFI), perfusion heterogeneity (e.g., an MFI-derived heterogeneity index, HI), and capillary density (e.g., functional capillary density, FCD). So far, technical limitations in image acquisition and labor-intensive computer-assisted manual image interpretation have prevented videomicroscopy from reaching the bedside [113]. Recently, Hilty and Ince introduced a fully automated IDF-compatible software platform (i.e., MicroTools) [114] able to collect all the parameters required to define the microcirculatory status according to an updated consensus paper on the measurement of sublingual microcirculation in the critically ill [112]. Using MicroTools, the same authors proposed a novel algorithm-based parameter (i.e., tissue RBC perfusion, tRBCp) that lumps all convective and diffusive microcirculatory determinants and promises to turn microcirculation monitoring into a readily available point-of-care modality to manage circulatory failure. Arguably, tRBCp is yet another reason to endorse a preload-sparing strategy in volume responsive patients as maximum recruitment of microcirculatory functional reserve is likely to precede CO maximization through full utilization of preload reserve [115]. In addition, dark field techniques incorporating multiwavelength oximetry could enrich tRBCp to provide an even more comprehensive snapshot of microcirculatory oxygen delivery [116].

Measures of tissue oxygenation provide an indirect assessment of the microcirculation. These mainly include tissue CO_2_ (tPCO_2_) and O_2_ (tPO_2_) tension and near-infrared spectroscopy (NIRS) for tissue oxygen saturation (StO_2_). Quasi-continuous static monitoring is standard with all three techniques but often challenging to interpret, given a low signal-to-noise ratio and a broad overlap between healthy and critically ill subjects. Accordingly, dynamic tests such as the oxygen challenge test (OCT) for transcutaneous tPO_2_ and vascular occlusion test (VOT) for thenar NIRS were devised to improve sensitivity and diagnostic yield. Failure to increase post-OCT tPO_2_ suggests inadequate tissue perfusion, hence a deteriorated microcirculation, and heralds increased mortality and organ failure [117]. Plotting StO2 against time during VOT allows more in-depth analysis. It first generates a de-oxygenation slope (DeO2) that correlates with local VO2 and, following occlusion release, a re-oxygenation slope (ReO2) that depends tightly on capillary reactivity, a marker of endothelial integrity [118]. VOT-derived parameters carry significant prognostic information according to several groups of authors [119,120,121]. By contrast, a recent systematic review proved that baseline StO2 values, but not VOT-derived parameters, predict mortality and could not assign a role to NIRS monitoring in treatment decisions based on current evidence [122].

To summarize, tissue-based parameters merely provide a global view on regional microcirculation as they fail to detect individual capillaries and provide relevant objective information on flow, perfusion heterogeneity, and vessel density. Thus, further research will have to address a yet non-standardized methodology and clinical integration with a fast-paced HVM technology close to becoming a reality for the everyday clinician.

Lastly, skin is a qualitative surrogate of impaired tissue perfusion that can be easily and quickly assessed through temperature (e.g., central-to-toe temperature gradient), perfusion (e.g., capillary refill time, CRT), and color (e.g., mottling) variations. Skin mottling score was shown to predict organ dysfunction and mortality in septic shock patients [123], even in prehospital settings [124]. The ANDROMEDA-SHOCK trial found that CRT-targeted resuscitation compared to lactate-targeted resuscitation did not significantly affect 28-day mortality but resulted in less organ dysfunction at 72 h [125]. A Bayesian analysis of these results reported improved mortality and lower SOFA scores at 72 h for the CRT-based group [126]. To date, CRT probably constitutes the most accessible route to probe microcirculation. Good inter-rater reproducibility, the ability to rapidly reflect ongoing therapy, and resource independence make CRT a reliable and integrative endpoint to apply during shock resuscitation.

## 4. Perspective

**Figure 6:**
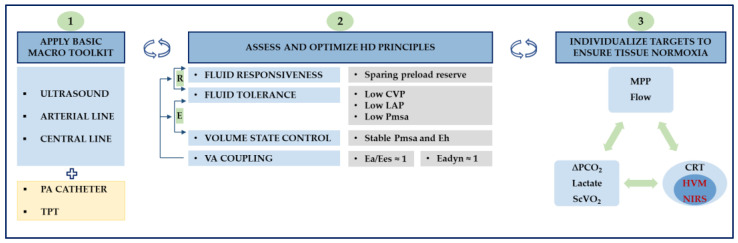
Conceptualized approach to shock management. Panel 1: a minimal hemodynamic toolkit is presented, encompassing ultrasound, arterial and central venous lines. Extended monitoring comprises pulmonary artery (PA) catheter and transpulmonary thermodilution (TPT). Panel 2: during active resuscitation (R), fluid responsiveness and fluid tolerance need simultaneous assessment, ensuring that: (1) preload reserve is spared, and (2) the minimum increase in intravascular pressures required to sustain adequate tissue perfusion is targeted to guard fluid tolerance. As a corollary, deterioration of fluid tolerance is poised to hamper tissue perfusion and hence requires resolution. During evacuation (E), fluid tolerance and volume state control need simultaneous assessment, ensuring that fluid removal rate is tuned to reach efficiency (lower CVP) and tolerance (preserved CO and MAP). Overall, stable Pmsa and Eh guarantee hemodynamic stability during evacuation phases. In preload independent states, higher removal rates and lower Pmsa can be achieved safely until a threshold is reached when further decreasing Pmsa would result in impaired Eh and low CO. VA coupling represents an energetic refinement of the cardiovascular state that may be superimposed regardless of phase. For practical reasons, Ea/Ees and Eadyn are set to 1 (See Table A1 and Table A2 for further discussion). Panel 3: An integrative approach encompassing macro- and microvascular targets is schematized, emphasizing that target individualization is paramount to improving outcomes. From a microcirculatory perspective, bedside clinicians must rely on clinical examination (i.e., CRT) until more objective monitoring (i.e., HVM) becomes available. CO, cardiac output; CRT, capillary refill time; CVP, central venous pressure; Ea, arterial elastance; Eadyn, dynamic arterial elastance; Ees, ventricular elastance; Eh, global heart efficiency; HVM, handheld vital microscope; LAP, left atrial pressure; MAP, mean arterial pressure; MPP, mean perfusion pressure; NIRS, near-infrared spectroscopy; Pmsa, mean systemic filling pressure analogue; ScVO2, central venous oxygen saturation; VA, ventriculoarterial; ∆PCO2, venous-to-arterial carbon dioxide partial pressure difference.

Priorities in tissue perfusion are likely to change with persistent states of shock. A strategy that integrates macro- and microcirculatory endpoints coupled with ultrasound may yield a personalized treatment plan with sound physiological roots (see Figure 6). Several systemic components, including ventricular energetics, closed-loop volume state control, and end-organ Doppler, are expected to undergo refinement or reformulation considering upcoming data.

Routine identification of microvascular phenotypes is mandatory to improve outcomes further. However, combining macro- and microhemodynamics remains a subject of intense debate. While some authors prioritize systemic endpoints but within an integrative approach [127], others promote a tissue-centered approach irrespective of macrocirculatory variables [128]. The pragmatic clinician realizes that these two options, far from being mutually exclusive, are, in fact, complementary. Because coherence is conserved in the early stages of shock, simply targeting macrohemodynamics may save time and resources without affecting outcomes. By contrast, deploying the same strategy in later stages of shock may be misleading and cause undue harm. Because incoherence increases with time, resuscitation should be oriented towards microcirculation in later stages.

## 5. Conclusions

The hemodynamic profiles of critically ill patients have varying degrees of complexity, susceptible to change over time. Precise hemodynamic profiling is paramount to ensure adequate supportive interventions and, ultimately, improved outcomes. Most patients fall within conventional recommendations, but many others fall outside and require an extended hemodynamic data set. Macrocirculatory and microcirculatory factors equally contribute to this division. Hence, a comprehensive circulatory workup framework combining a set of hemodynamic principles with macro- and microcirculatory resuscitative endpoints was presented, aiming to explore critically ill patients across all shock trajectories. Future research is warranted to encourage and further define its use at the bedside.

## Data Availability

Not applicable.

## Appendix A

**Table A1:** Questions to be answered by a minimal toolkit consisting of ultrasound, arterial and central venous lines. Adapted with permission [60].

Cardiac Pathology	Tools
What is the CO/SV? Does CO/SV fit the clinical context?	Echocardiography 2D-imaging Doppler
2. Is there a hemodynamically consequential pericardial effusion?	
3. Are there features of LV systolic dysfunction?	
4. Are there LV regional wall motion abnormalities?	
5. Are there features of LVOT obstruction?	
6. Are there features of LV diastolic dysfunction/increased LAP?	
7. Are there features of RV dysfunction or raised PAP?	
8. Is there any severe functional or organic valvular heart disease?	
9. Are there features of volume overload?	
10. Is there a cardiac source of infection (e.g., endocarditis)?	
11. Is there a hyperkinetic profile? ^a^ If affirmative, is it caused by hypovolemia or vasoplegia?	Echocardiography (CO), arterial line (MAP) and central venous line (CVP)
**Hemodynamic Principles** **Cardiovascular Performance and Functional Reserve**	**Tools**
Is CO/SV responsive to fluid administration?	Echocardiography Doppler (i.e., LVOT VTI)
2. Is fluid tolerance at risk? Is there venous congestion?	Ultrasound Lung score Doppler (renal, hepatic and portal vein flow pattern) IVC diameter
3. ^b^ Is there abnormal left VA coupling?	Echocardiography (SV, LVEF, PET, TET) and arterial line (SAP, DAP)
4. ^c^ What is the volume state (Pmsa)? What is the global heart efficiency (Eh)? What is the volume efficiency (Evol)?	Echocardiography (CO), arterial line (MAP) and central venous line (CVP)

^a^ Hypovolemia and vasoplegia (i.e., relative hypovolemia) are associated with reduced stressed blood volume and venous return and exhibit indistinguishable echocardiographic features. Clinical context may guide in setting these two conditions apart. In addition, vasomotor tone (i.e., SVR) is typically low in vasoplegia and could be easily computed at the bedside: 80 × (MAP − CVP) = CO_ECHO_ × SVR. ^b^ Left VA coupling, defined as the ratio of arterial elastance (Ea) to LV end-systolic elastance (Ees), can be computed at the bedside using Chen’s method [81], recently available as a free mobile application [85]. Also, the dynamic arterial elastance (Eadyn) can be computed as pulse pressure variation (PPV) over stroke volume variation (SVV), preferably with two independent signals (i.e., arterial line for PPV and echocardiography for SVV). A practical single cut-off of 1 could be used to discriminate the pressor response to both fluid loading (i.e., MAP increases after fluid in preload dependent patients) and vasopressor weaning (i.e., MAP decreases after norepinephrine dose reduction) [91,93]. ^c^ Assessment of the volume state rests on the determination of Pmsa according to the mathematical model of Parkin and Leaning. Pmsa = 0.96 × CVP + 0.04 × MAP + c × CO_ECHO_, where c is an individual anthropometric constant (i.e., dependent on age, height, and weight). Eh = (Pmsa − CVP)/Pmsa. Eh (global heart efficiency) is a dimensionless ratio between zero and one, where zero represents “no flow” and one represents ideal heart performance. The typical Eh range in critically ill patients is between 0.3 and 0.5. Evol = ∆(Pmsa − CVP)/∆Pmsa. Evol (volume efficiency) is a scalar and continuous measure of the efficiency of a preload change (e.g., after PLR) to increase CO. Preload responsiveness is incrementally associated with Evol ≥ 0.35 after PLR; Evol range, [0; 1] [99]. CO, cardiac output; CVP, central venous pressure; DAP, diastolic arterial pressure; Eh, global heart efficiency; IVC, inferior vena cava; LAP, left atrial pressure; LV, left ventricle; LVEF, ejection fraction of left ventricle; LVOT, left ventricular outflow tract; MAP, mean arterial pressure; PAP, pulmonary artery pressure; PET, pre-ejection time; PLR, passive leg raising; Pmsa, mean systemic filling pressure analogue; RV, right ventricle; SAP, systolic arterial pressure; SV, stroke volume; SVR, systemic vascular resistance; TET, total ejection time; VA, ventriculoarterial; VTI, velocity time integral.

**Table A2:** Decision tree algorithm to help integrate VA coupling into routine hemodynamic therapy according to Guarracino et al. [84].

VA Ratio	VA Components	Proposed Therapy
$\frac{Ea}{Ees}$ < 1	Ea < 2 and Ees > 2	Vasopressor
	Ea < 2 and Ees < 2	Inotrope
$\frac{Ea}{Ees}$ > 1	Ea > 2 and Ees < 2	Inodilator
	Ea >> 2 and Ees ≈ 2	Vasodilator

Ea, arterial elastance; Ees, left ventricular end-systolic elastance; VA, ventricular-arterial.
